# Prevalence of Gastrointestinal Helminthiasis in Horses and Donkeys of Hawassa District, Southern Ethiopia

**DOI:** 10.1155/2021/6686688

**Published:** 2021-05-07

**Authors:** Mesfin Mathewos, Dawit Girma, Haben Fesseha, Metages Yirgalem, Eyob Eshetu

**Affiliations:** ^**1**^ School of Veterinary Medicine, Wolaita Sodo University, P.O. Box: 138, Wolaita Sodo, Ethiopia; ^2^School of Veterinary Medicine, Haramaya University, PO. Box: 138, DireDawa, Ethiopia

## Abstract

**Background:**

Gastrointestinal helminth parasite infection is a major influencing factor against the profitability of working equines all over the world.

**Methods:**

A cross-sectional study was conducted to determine the prevalence of gastrointestinal tract (GI) helminths and assess the associated risk factors in donkeys and horses in the Hawassa district. A total of 214 fecal samples were collected from randomly selected equines (112 donkeys and 102 horses) and examined for the presence of eggs of GI helminths using standard coprological techniques.

**Results:**

According to the current study, the prevalence of GI helminths in equine was 78.5% (168/214) and the highest prevalence was reported in donkeys 92% (103/112) as compared to horses 63% (65/102). Out of 168 positive samples, the different species of parasites were identified, namely, *Strongyle* species 120 (56.1%), *Strongyloides westeri* 76 (35.5%), *Parascaris equorum* 54 (25.2%), *Anoplocephala perfoliata* 34 (15.8%), *Oxyuris equi* 20 (9.3%), *Fasciola hepatica* 18 (8.8%), *Gastrodiscus* species 12 (5.6%), and *Dictyocaulus arnfieldi* 4 (1.8%). There was a strong association between the prevalence of GI helminths and factors such as the species of equine, body condition scores, and feed type (*p* < 0.05). However, no significant association (*p* > 0.05) was observed between the prevalence of GI helminths and putative risk factors such as age, sex, housing, and water source. The coproculture performed on 30 pooled fecal samples revealed that *Cyathostomes species*, *Strongylus vulgaris*, and *Strongylus edentatus* were the major helminth parasites of donkeys and horses.

**Conclusion:**

The frequency of gastrointestinal helminths in equine species was high, especially in donkeys. Hence, strategic deworming using broad-spectrum antihelminthic drugs and a rotational grazing program should be implemented to control and prevent the diseases.

## 1. Introduction

Donkeys (*Equus asinus*) and horses (*Equus caballus*) are among the early domesticated equines that have been around as long as 3000 years ago [1]. The world equine population is estimated at about 59 million horses, 52 million donkeys, and 15 million mules [2]. More than 97% of the world's donkey and mule populations and over 72% of the world's horse population are found in developing countries and specifically kept for the draft purpose [3,4]. In Africa, the donkey and Horse population are estimated to be 13.7 and 9 million, respectively [5], whereas Ethiopia has more than 8 million donkeys, the second-largest donkey population in the world next to China, more than 2 million horses, and over 350,000 mules [6]. In Ethiopia, the low level of development of the road transport network and the rough terrain of the country makes equines the most valuable, appropriate, and affordable pack animals under the smallholder farming system [3, 7].

Horses, donkeys, and mules are extensively used, particularly in rural socioeconomic activities [4, 8]. Horses are transport animals, used for riding as well as for rural and urban transport. Other important working animals include mules that are mainly used in the hilly areas for packing and riding and playing an important role in rural and periurban communities providing transport at low cost. Donkeys are mainly used to breed mules and light transport in the mountains [3, 4, 9]. Despite all these, donkeys and horses are prone to several infectious and noninfectious diseases. The care and management provided for equines are very low, and relatively less attention is given to donkeys which is far below to what it deserves [3, 10, 11]. Among these diseases, they are hosts to a great number of gastrointestinal parasite species [12].

Gastrointestinal parasitism is identified as one of the most important problems for equids in developing countries [13], and it affects the health and working performance of donkeys and horses worldwide. Gastrointestinal helminths cause various degrees of damage depending on the species and nutritional and immune status of equines. They decrease the performance and productivity in the animals, mainly the reduction of body weight or failure to gain weight, or even increase the mortality in acute cases [14].

Studies conducted in Ethiopia and Mexico estimate the prevalence of endoparasite infections at over 90% in horses [15–17] and over 80% in donkeys [18–20]. The most commonly identified gastrointestinal helminths of equines in different parts of the country include *Strongyle*, *Cyathostomess*, *Triodontophorus species*, *Strongyloides westeri*, *Parascaris equorum*, *Dictyocaulus arnfieldi*, *Oxyuris equi*, *Gastrodiscus*, and *Fasciola* species [21]. However, information regarding the prevalence and type of internal parasites affecting equines has not been recorded well in the southern part of Ethiopia in general and in the Hawassa district in particular. Therefore, this study was conducted to determine the prevalence of gastrointestinal helminth parasites and associated risk factors in horses and donkeys in the study area.

## 2. Materials and Methods

### 2.1. Study Area

**Figure 1:**
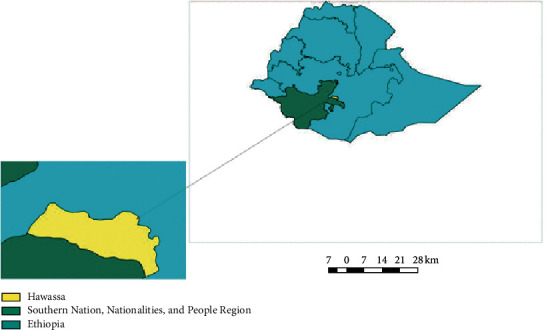
Geographical location map of Hawassa [23].

The study was carried out from October 2019 to May 2020 in and around Hawassa. Hawassa is located 273 km south of Addis Ababa via Bishoftu, 130 km east of Sodo, and 75 km north of Dilla at a latitude of 7°04′N and a longitude 38°31′E on the escarpment of the Great Rift Valley. The altitude ranges from 1650 to 1700 m above sea level. The mean annual rainfall and temperature are 900–1100 mm and 27°C, respectively [22] (Figure 1).

### 2.2. Study Animals and Design

A cross-sectional study was conducted on 214 randomly selected indigenous breeds of donkeys and horses to determine the prevalence and potential risk factor of gastrointestinal helminths using the coprological examination. Factors such as species, sex, age, body condition score (BCS), feeding, and water were considered. The study includes horses and donkeys of all age groups and both sexes, kept under extensive management systems. The age of the selected horse and donkeys was determined from birth records and dentition characteristics [24] accordingly, and equines were grouped into three age categories: from 1 to 3 years of age were classified as young, 3–10 years as an adult, and those beyond 10 years as old. Body condition scoring (BCS) of the horses and donkeys were estimated based on the guides described by Elisabeth [25] and classified into poor, medium, and good body conditions.

### 2.3. Sample Size Determination

The sample size required for this study was determined according to the work of Thrusfield [26]. There was a previous work conducted in this study area where 96% and 97% prevalence was taken for donkey and horses, respectively, as the expected prevalence for the determination of sample size. The other determinants considered in sample size determination were 95% confidence interval and 5% desired absolute precision. Hence, the sample size is estimated as

(1) $$\begin{array}{c} N=\frac{1.96^{2}P_{\exp}\left(1-P_{\exp}\right)}{d^{2}}, \end{array}$$

where *N* = required sample size, P_exp_ = expected prevalence, *d* = absolute precision, and *Z* = 1.96 at 95% level of confidence. Thus, a total of 214 cattle were included in the current study.

### 2.4. Study Methodology

#### 2.4.1. Fecal Sample Collection and Handling

Fecal samples were collected directly from the rectum or sometimes from freshly passed feces using plastic gloves in clean plastic bags. Along with sampling, date, name, identification number, age, sex, BCs, feed type, housing condition, and water source were recorded. Then, all fecal samples were transported to the Hawassa University parasitology laboratory for further fecal examination from the selected site by using iceboxes and analyzed in the regional laboratory. Fecal samples were examined using standard parasitological techniques (flotation, sedimentation, and modified Baermann) and examined using 10x and sometimes 40x magnification power [27].

The larvae were then identified based on the shape and number of gut cells, relative size, and shape of the larvae's tail. The floatation fluid used in this study was a supersaturated solution of sodium chloride (NaCl) salt prepared in the laboratory according to the procedures given by Zajac and Conboy [28]. Moreover, the eggs were identified using ova identification keys [29, 30].

### 2.5. Data Management and Statistical Analysis

The collected data from the field were entered into a Microsoft Excel 2016 spreadsheet and analyzed using the STATA version 13 software program. The prevalence was calculated as the number of animals having parasites, divided by the total number of animals examined. The association between the risk factors and the outcome variables was assessed using the *chi*-square (*X*^2^) test. For all analysis, a *p* value less than 0.05 was considered as significant.

## 3. Results

### 3.1. Prevalence of Gastrointestinal Helminths in Donkeys and Horses

The overall prevalence of helminth parasites of equine in the study area was found to be 78.5% (168/214) with a prevalence of 91.9% (103/112) and 63.73% (65/102) in donkeys and horses, respectively (Table 1).

### 3.2. Species Distribution of Gastrointestinal Helminths in Donkeys and Horses

**Figure 2:**
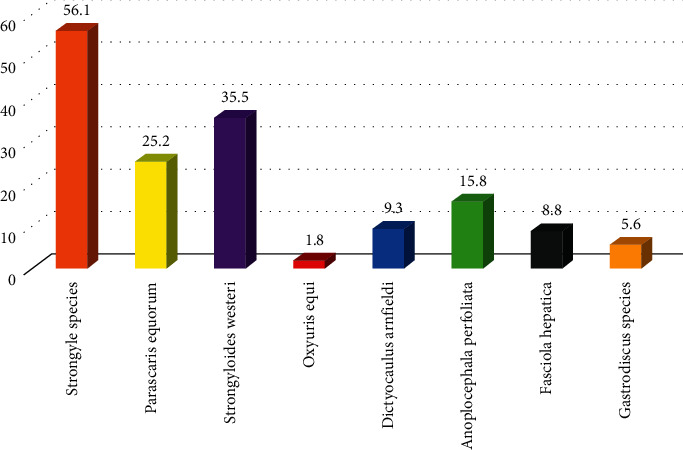
Proportion of different species of GI helminths identified in the study area.

The present study revealed that eight different species of GI helminths were identified in this study area. *Strongyle* species 120 (56.1%), *Parascaris equorum* 54 (25.2%), *Strongyloides westeri* 76 (35.5%), *Oxyuris equi* 20 (9.3%), *Dictyocaulus arnfieldi* 4 (1.8%), *Anoplocephala perfoliate*, 34 (15.8%), *Fasciola hepatica* 18 (8.8%), and *Gastrodiscus* species 12 (5.6%) (Figure 2).

In donkeys, the prevalence of *Strongyle* species, *Strongyloides westeri*, and *Parascaris equorum* was 66%, 50%, and 34.8%, respectively, while in horses, the prevalence of *Strongyle* species, *Strongyle westeri*, and *Anoplocephala perfoliata* was found to be 45.1%, 19.6%, and 15.7%, respectively. In both donkeys and horses, *Strongyle* species were found as the most prevalent GI helminth parasites in the study area. However, *Dictyocaulus arnfieldi* were the least prevalent parasites in donkeys with no observation of this parasite in horses during the study period (Table 2).

### 3.3. Association of Equine Gastrointestinal Helminths in Relation to Different Risk Factors

In this study, there was a significant association (*p* < 0.05) of the prevalence of GI parasites with species of animal, body condition score, and feed type. However, age, sex, housing, and water have no significant association (*p* > 0.05) with the prevalence of GI parasites (Table 3).

### 3.4. Fecal Culture Output

In the current study, fecal culture was conducted to identify the larvae of nematodes. *Strongylus vulgaris* (36.6%) and *Cyathostomins* species (36.6%) were recorded in a higher proportion in horses than in donkeys, whereas a higher proportion of *Strongylus edentatus* (13.3%) was recovered from donkeys than horses (Table 4).

## 4. Discussion

Gastrointestinal parasite infection directly affects the health and production of working equines, which contributes to the reduction in their work output and, ultimately, in the income of the owner and the community [31, 32]. In this study, the overall prevalence of gastrointestinal helminth was 78.5%, where a higher prevalence of GI parasitism was recorded in donkeys (91%) than horses (63%). This finding was lower as compared to the reports of Mezgebu et al. [14], who reported 92.71% in Gondar, Ethiopia, and Debra et al. [31], who reported 88.8% in Guder, west Shewa, Ethiopia, Takele and Nibret [33], who reported 88.2% in Bahirdar, Chemeda et al. [34], who reported 94.0% in Ambo, Ayele et al. [11], who reported 98.2% in Dugda Bora district, Tolossa and Ashenafi [7], who reported 92.71% in Arsi-Bale highland, Gulima [35], and Seyoum et al. [36], who reported 84.4% in Awi Zone and around Shashemane.

The prevalence of GI helminths reported in the same study area by Ibrahim et al. [37] and Berhanu et al. [38] was 96.9% and 97.9% in donkeys and horses. This difference might be due to the nutritional status of the animal in the respective study area which can influence the level of immunity to be infected by the parasite. Additionally, it could be affected by the deworming habit of the equine and accessibility to the veterinary clinic. Also, there was a statistically significant difference (*p* < 0.05) with the prevalence of GI parasites and feed which was in agreement with the work of Mezgebu et al. [14].

In this study, a relatively higher prevalence of gastrointestinal parasites was recorded in donkeys (97.1%) than horses (81%). This was comparable with the previous finding of Debere et al. [31], who reported higher gastrointestinal parasites in donkeys (95.4%) than horses (89.7%), and Tolossa and Ashenafi [7] in donkeys (97.13%) than in horses (80.95%). The observed higher parasitism in donkeys could be attributed to the fact that less attention was given to these animals. Also, it might be related to the feeding practices as all donkeys under the study were at free grazing that they have a high chance of ingesting large amount of gastrointestinal parasite eggs and larvae.

The prevalence of *Strongyle* species in horses was 45.1% in the current study which was lower than the previous reports described by Mezgebu et al. [14] who reported 66.7% prevalence in Gondar Town and Uslu and Guçlu [39] who reported 100% in Konya, Turkey. The lower prevalence in the present study could be because all horses of this study were less exposed and, in some cases, totally restricted from pasture.

The prevalence of *Strongyle*-type infestation in donkeys was 66.07%. This result disagreed with studies conducted in different areas with 87.8% by Mezgebu et al. [14] in Gondar Town, 76% by Tesfu et al. [12] in Hawassa, 80.2% by Asefa and Dulo [40] in Bishoftu, 79.7% by Abdulahi et al. [41] in Jigjiga, 81% by Uslu and Guçlu [39] in Konya, Turkey, and 99.5% by Naramo et al. [42] in Alage, south-western Ethiopia.

The current prevalence of *Strongyloides westeri* in the horses was 19.6% which was in agreement with the work of Ibrahim et al. [37] who reported 20% prevalence in Hawassa town, Southern Ethiopia. However, a higher prevalence was observed as compared with the previous report by Belete and Derso (2015) in Mekelle and Uslu and Guçlu [39] in Konya who reported the prevalence of 0.8% and 7.2%, respectively. This might be because patent infections develop primarily by transmammary infection of foals of *Strongyloides westeri* in the study area. Its prevalence in donkeys was 50.0% which is higher than in the report of Uslu and Guçlu [39] in Konya, Getahun and Kassa [43] in Tenta woreda, Gebreyohans et al. (2017) in and around Mekelle, and Ibrahim et al. (2011) in Hawassa with the prevalence of 12.3%, 9.5%, 2%, and 20%, respectively.

The prevalence of *Parascaris* in horse under this study was 14.7% which was lower than in the reports of Berhanu et al. [38] (55.8%) in Hawassa town, Mezgebu et al. [14] (43.8%) in Gondar town, 32.8% by Chemeda et al. [44] in and around Ambo Town, and 25% by Asefa and Dulo [40] in Bishoftu town. On the other hand, the prevalence of *Parascaris* in the present study was higher than in the previous report by Tesfu et al. (2014) in Hawassa Town, Uslu and Guçlu [39] in Konya, and Belete and Derso [45] in Mekelle with the infestation rate of 4.6%,10.8%, and 1.8%, respectively.

In donkeys, the prevalence of *Parascaris* was found to be 34.8% which was higher than the prevalence reported by Getahun and Kassa [43] (11.2%) in Tenta woreda, 6.4% by Gebreyohans et al. [46] in and around Mekelle, 26.2% by Tesfu et al. [12] in Hawassa Town, 15.1% by Asefa and Dulo, [40] in Bishoftu Town, and 9.8% by Uslu and Guçlu [39] in Konya. Conversely, it was lower than in reports described by Naramo et al. [42], Ibrahim et al. [37], Abdulahi et al. [41], and Mezgebu et al. [14] who represented a prevalence of 53.6%, 52.8%, 44.8%, and 42.3% in donkeys, respectively. All this difference might be because this parasite was found to be persistent generally in low numbers in the pasture despite their parasite control programs applied in recent years.

The current prevalence of *Oxyuris equi* in horses was 9.8% which was nearly in agreement with the prevalence reported by Uslu and Guçlu [39] (8.8%) in Konya, 8.7% by Asefa and Dulo [40] in Bishoftu, 8.8% by Belete and Derso [45] in Mekelle, and 11.2% by Chemeda et al. [44] in and around Ambo Town. However, it was higher than reports described 0.4% by Mezgebu et al. [14] in Gondar and 1.8% by Uslu and Guçlu [39] in Konya. In the current study, the prevalence of *Oxyuris equi* in donkeys were 8.9%, which showed a significantly higher prevalence as compared with other studies mentioned by Mezgebu et al. [14] in and around Gondar, Asefa, and Dulo [40] in Bishoftu Town, Uslu and Guçlu [39] in Konya, and Getahun and Kassa [43] in Tenta Woreda with the prevalence of 4.3%, 5.2%, 1.2%, and 2.7%, respectively. This is somewhat conflicting and this could be due to compromised immune responses relating to concurrent disease, but it needs further investigation.

The current prevalence of Fasciolosis in horses was 13.7%, which disagreed with the work of Ayana et al. [47] who reported 24% prevalence in Holeta town. Although the prevalence of Fasciolosis in donkeys were found to be 4.5% which was nearly agreed with the results of Abdulahi et al. [41] from Jigjiga, Gebreyohans et al., [46] in and around Mekelle, and Getahun and Kassa [43] in Tenta Woreda who reported 1.6%, 2.2%, and 2% prevalence, respectively. However, the higher prevalence was reported by Uslu and Guçlu [39] in Konya and Mezgebu et al. [14] in Gondar Town who reported 6.2% and 5.7% prevalence, respectively.

The prevalence of *Anoplocephala perfoliata* in horses was 15.7% which is higher than the prevalence reported in 2.7% by Uslu and Guçlu [39], 2% by Belete and Derso [45], and 1.6% by Chemeda et al. [44]. Also, the prevalence of *Anoplocephala perfoliata* in donkeys was 16.1% which showed a higher infestation rate as compared with 6.2% by Uslu and Guçlu [39], 2.6% by Getahun and Kassa [43], and 2.7% by Gebreyohans et al. [46].

In the present study, *Dictyocaulus arnfieldi* was not observed in the horse. However, its prevalence in donkeys was 3.6% which agreed with the previous report by Ibrahim et al. [37], who reported the 3.6% prevalence in Hawassa town, southern Ethiopia.

The current prevalence of *Gastrodiscus* species was 6.8% and 4.5% in horses and donkeys, respectively. These results were higher than Mezgebu et al. [14] who reported 2.9% and 3.6%, respectively, in Gondar. However, the result was lower than the prevalence reported by Ayana et al. [47] in Holeta Town, Oromia, Central Ethiopia, who reported 13.7% in horses and 10.6% in donkeys.

From the coproculture study, the present study revealed that *Cyathostomins* species was highly prevalent than *S*. *vulgaris* and *S*. *edentatus* with a prevalence of 43.3% and 63.3%; 26.6% and 36.6%; and 13.3% and 10% in donkeys and horses, respectively. This higher prevalence of cyathostomins species in the present studies was in agreement with Uslu and Guçlu [39].

## 5. Conclusions and Recommendations

Internal parasites are important health problems of equines in the study area with an overall prevalence of 78.5%, especially the highest in donkeys with 92% followed by horses (63%). Factors such as species, body condition, and feed type were strongly associated (*p* < 0.05) with the occurrence of the disease. *Strongyle* species (56.1%), *Parascaris equorum* (25.2%), *Strongyloides westeri* (35.5%), *Oxyuris equi* (9.3%), *Dictyocaulus arnfieldi* (1.8%), *Anoplocephala perfoliate*, (15.8%), *Fasciola hepatica* (8.8%), and *Gastrodiscus* species (5.6%) were the most dominant gastrointestinal parasites affecting horse and donkeys in the study area. The coproculture investigation revealed that *Strongylus vulgaris* (36.6%) and *Cyathostomins* species (36.6%) were recorded commonly in horses as compared to donkeys, whereas a higher proportion of *Strongylus edentatus* (13.3%) was recovered from donkey than horses.

In conclusion, in order to minimize losses attributed to the equine internal parasites in the area, equine owners should be informed of the economic importance and methods of control and prevention of helminths of equines through management improvements. Furthermore, regular and strategic deworming programs with efficacious anthelmintics should be carried out regularly.

## Figures and Tables

**Table 1:** Overall prevalence of GI helminth parasites in horses and donkeys.

Animal species	No. of examined animals	No. of positives	Proportion (%)
Equines (horse and donkey)	214	168	78.5
Donkeys	112	103	91.9
Horses	102	65	63.7

**Table 2:** Distribution of species of GI helminths identified from donkeys and horses.

Parasites species	Species of animal			
	Donkeys (*n* = 112)		Horses (*n* = 102)	
	No. of positives	Prevalence (%)	No. of positives	Prevalence (%)
*Strongyle* species.	74	66	46	45.1
*Parascaris equorum*	39	34.8	15	14.7
*Strongyloides westeri*	56	50	20	19.6
*Oxyuris equi*	10	8.9	10	9.8
*Dictyocaulus arnfieldi*	4	3.5	0	0.0
*Anoplocephala perfoliata*	18	16.1	16	15.7
*Fasciola hepatica*	5	4.5	14	13.7
*Gastrodiscus* species	5	4.5	7	6.8

**Table 3:** Prevalence GI parasites in association with risk factors of equine.

Variable	Category	Total	Positive	*X* ^2^ value	*p* value
Species	Donkey	112	103	25.23	0.0001
	Horse	102	65		
Body condition score	Good	60	47	8.26	0.016
	Medium	94	81		
	Poor	60	40		
Age	Young	36	27	0.32	0.575
	Adult	178	141		
Sex	Male	157	126	1.07	0.301
	Female	57	42		
Feed type	Roughage	62	43	4.33	0.037
	Concentrate	152	125		
Housing condition	Good	149	113	2.07	0.151
	Poor	65	55		
Water source	Tap water	65	55	2.07	0.151
	Well water	149	113		

**Table 4:** Frequency of GI helminths in donkeys and horses after fecal culture.

Species of parasites	Species of animal			
	Donkeys (*n* = 30)		Horses (*n* = 30)	
	No. of harvested larvae	Prevalence (%)	No. of positives	Prevalence (%)
*Strongylus vulgaris*	8	26.6	11	36.6
*Strongylus edentatus*	4	13.3	3	10
*Cyathostomins* species	13	43.3	19	63.3

## Data Availability

The datasets used and analyzed during the current study are available from the corresponding author on request.

## Abbreviations

GI: Gastrointestinal.
